# Genomic characterization and detection of potential therapeutic targets for peritoneal mesothelioma in current practice

**DOI:** 10.1007/s10238-024-01342-y

**Published:** 2024-04-20

**Authors:** Job P. van Kooten, Michelle V. Dietz, Hendrikus Jan Dubbink, Cornelis Verhoef, Joachim G. J. V. Aerts, Eva V. E. Madsen, Jan H. von der Thüsen

**Affiliations:** 1 Department of Surgical Oncology and Gastrointestinal Surgery, Erasmus MC Cancer Institute, P.O. Box 2040, 3000 CA Rotterdam, the Netherlands; 2 Department of Pathology, Erasmus MC, Rotterdam, the Netherlands; 3 Department of Pulmonary Oncology, Erasmus MC Cancer Institute, Rotterdam, the Netherlands

**Keywords:** Peritoneal mesothelioma, Next generation sequencing, Targeted therapy, Foundation medicine

## Abstract

Peritoneal mesothelioma (PeM) is an aggressive tumor with limited treatment options. The current study aimed to evaluate the value of next generation sequencing (NGS) of PeM samples in current practice. Foundation Medicine F1CDx NGS was performed on 20 tumor samples. This platform assesses 360 commonly somatically mutated genes in solid tumors and provides a genomic signature. Based on the detected mutations, potentially effective targeted therapies were identified. NGS was successful in 19 cases. Tumor mutational burden (TMB) was low in 10 cases, and 11 cases were microsatellite stable. In the other cases, TMB and microsatellite status could not be determined. *BRCA1 associated protein 1 (BAP1)* mutations were found in 32% of cases, *cyclin dependent kinase inhibitor 2A/B (CDKN2A/B)* and *neurofibromin 2 (NF2)* mutations in 16%, and *ataxia-telangiectasia mutated serine/threonine kinase (ATM)* in 11%. Based on mutations in the latter two genes, potential targeted therapies are available for approximately a quarter of cases (i.e., protein kinase inhibitors for three *NF2* mutated tumors, and polyADP-ribose polymerase inhibitors for two *ATM* mutated tumors). Extensive NGS analysis of PeM samples resulted in the identification of potentially effective targeted therapies for about one in four patients. Although these therapies are currently not available for patients with PeM, ongoing developments might result in new treatment options in the future.

**Supplementary Information:**

The online version contains supplementary material available at 10.1007/s10238-024-01342-y.

## Introduction

Peritoneal mesothelioma (PeM) is an aggressive tumor, arising from the peritoneum [1]. It comprises about ten to fifteen percent of all mesotheliomas, thereby being the second most common variant after pleural mesothelioma [2]. Due to its rarity and non-specific symptoms, it is often diagnosed at an advanced stage. Currently the best available treatment is a combination of cytoreductive surgery (CRS) with hyperthermic intraperitoneal chemotherapy (HIPEC) [3]. Unfortunately, most patients experience disease recurrence, even after complete cytoreduction. Adding (neo)adjuvant systemic chemotherapy to the treatment does not result in improved disease-free, or overall, survival [4], and only a small proportion of patients are eligible to undergo surgical treatment, while there is a lack of effective systemic treatment options [5].

Because PeM is so rare, it is especially hard to gather (randomized) evidence on the effect of new therapeutics. The heterogeneity of the tumor further complicates this research. Personalized strategies, based on tumor molecular characteristics, could be promising [6]. One approach is to identify potentially targetable mutations, which can be treated with readily available therapies. However, data on the mutational landscape of PeM have long been lacking. Recently, several studies have been published that provide more insights in the mutational profile of PeM [7–11]. These data could aid to identify new treatment options for patients with PeM. Preferably, these treatments are already registered for the treatment of (other) cancers, but currently there are also clinical trials that include patients based on tumor molecular characteristics rather than cancer type or location [12–14].

Foundation Medicine (FMI) offers a platform (Foundation One® CDx (F1CDx)) for next generation sequencing (NGS) of formalin fixed paraffin embedded (FFPE) tumor samples, which are often the only material available from diagnostic biopsies. The platform assesses a total of 360 genes that are known to be somatically mutated in solid tumors [15]. It also provides a genomic signature, by assessing tumor mutational burden (TMB) and microsatellite (in)stability (MSS/MSI). To evaluate the value of genomic characterization in patients with PeM in current daily practice, we performed broad targeted NGS on tumor biopsies from 20 patients who were referred to the Erasmus MC Cancer Institute from 2018 to 2021.

## Methods

### Patient selection and data handling

From 2018 to 2021, 41 PeM patients were referred to the Erasmus MC Cancer Institute in Rotterdam, a Dutch mesothelioma expert center. From these 41 patients, we identified 23 patients for whom excess tumor tissue was available and who provided permission to use this tissue for research purposes. NGS by Foundation Medicine (FMI) F1CDx was available for 20 tumor samples. To maximize the chance of finding new significant mutations, we further selected the patients based on sex, age and lack of asbestos exposure, thus enriching the cohort for females and younger patients [16]. All data were collected and managed according to the latest European privacy regulations (General Data Protection Regulation (GDPR), EU 2016/679). The study was approved by the EMC local ethics committee (MEC 2018-1286).

### The foundation one® CDx assay

F1CDx uses DNA, acquired from FFPE tissue samples, for NGS of solid tumors. A comprehensive method description can be found in the technical information [15]. The assay is able to detect alterations in a total of 324 different genes, and another 36 introns of genes that are involved in rearrangements. Mutations in these genes and genetic rearrangements are known to occur in solid tumors and might be drive alterations for oncogenesis. Moreover, many of these mutations are susceptible to targeted therapies. A full list of included genes/rearrangements is rendered in the supplementary data (supplementary Table 1). The assay also determines the genetic signature of the tumor, by providing microsatellite status (MSI), and tumor mutational burden (TMB). MSI status is determined by genome wide analysis of 95 microsatellite loci. The assay report that is provided by Foundation One® also includes suggested (targeted) therapies or clinical trials for individual patients, based on latest available clinical evidence and an up-to-date overview of current clinical trials that include patients based on certain mutations.

## Results

### Patient and tumor characteristics

Broad targeted NGS on tumor biopsies from 20 individual patients was performed. Unfortunately, this resulted in one sample failure, leaving 19 samples to be fully analyzed. Table 1 provides a comprehensive overview of patient and disease characteristics per patient. The patients included in the study had a median age of 54 years (IQR 48–63), and three (15%) were female. Epithelioid morphology was most common, observed in 18 patients (90%), while sarcomatoid and biphasic morphology were each present in one patient (5%), as determined by an experienced subspecialist pathologist (JT) by histological analysis of hematoxylin/eosin (H&E) stained sections of FFPE tissue. A minority of patients (40%) had been (occupationally) exposed to asbestos in the past. The median peritoneal cancer index (PCI), a measure used to determine the extent of peritoneal disease, was 39 (IQR 31–39) [17]. Most patients (80%) presented with ascites at time of diagnosis and two patients (10%) had nodal dissemination. The Ki67 (or MIB) index reflects the percentage of proliferating cells and is a known prognostic indicator for PeM patients. Median Ki67 index was 8% (IQR 5–19%); while 11 tumors (58%) had a Ki67 index below 10% and eight tumors (42%) had a Ki67 index equal to or greater than 10%. Germline mutation analysis was performed in five out of 20 patients, of whom two patients were carrier of a BRCA associated protein 1 (*BAP1*) germline mutation.

**Table 1:** Overview of patients and tumor characteristics

Patient	Sex	Age at diagnosis	Histological subtype	Lymph node metastases	Ki67 (%)	PD-L1 (%)	BAP1 germline	BAP1 IHC	MTAP IHC	Tumor purity (%)	Gene alterations	VAF (%) ^a^	TMB (muts/mb)	MS status	Mutations of unknown significance	Approved targeted therapies ^b^	Targeted therapies investigated in clinical trials ^c^
1	F	36	Epithelioid	No	3	UND	No	positive	positive	10,1	WT1 splice site 1340-1G > A	2,3	UND	UND	AR, EP300, GRM3, LTK, NTRK1, PIK3C2B, SETD2	None	None
2	M	39	Epithelioid	No	4	UND	Yes	loss	positive	60,0	BAP1 K61fs*11	77,5	4	MSS	IRF2, NF1, NOTCH3, POLE, TBX3	None	EZH2 inhibitors
3	M	48	Epithelioid	No	10	UND	UND	loss	positive	48,7	BAP1 loss PIK3CA amplification SOX2 amplification ^#^ ATR rearrangement exon 39 EPHB1 amplification ^#^ PBRM1 loss (exons 13–30) PRKCI amplification TERT promotor -124C > T	34,8	0	MSS	CTNNA1, KMT2A, MAP3K13, PRKCI, RAR, TERC, TIPARP	None	EZH2 inhibitors PARP inhibitors PI3K inhibitors mTOR inhibitors
4	M	51	Epithelioid	No	15	UND	No	positive	inconclusive	37,0	NF2 Q212* CDKN2A/B loss	22,5	0	MSS	ARID1A, ESR1, MDM4	mTOR inhibitors	FAK inhibitors mTOR inhibitors CDK4/6 inhibitors Pan-ERBB inhibitors
5	M	55	Epithelioid	No	7,5	5	UND	loss	UND	71,8	FLT3 N841T^♱^ PBRM1 rearrangement exon 26	1,5	0	MSS	CXCR4, FANCA, HGF	None	None
6	M	57	Epithelioid	No	2	UND	UND	positive	positive	57,7	CDH1 R732Q ^♱^ MSH6 F1245fs*31^d^ MUTYH G382D TP53 R175H ^♱^ TP53 R273C ^♱^ TP53 R158H ^♱^ TP53 R273H	2,6 62,8 47,9 1,1 1,1 1,4 26,6	1	MSS	ALK, MSH3, ERRFI1, PPP2R2A, MDM4, ROS1, MEN1	None	None
7	M	61	Epithelioid	No	10	UND	UND	UND	UND	10,1	BAP1 splice site 554_580 + 12del39, Y33fs*1	5,1 5,6	UND	UND	CSF1R, KDR, POLE	None	EZH2 inhibitors
8	F	62	Epithelioid	No	4	UND	UND	loss	UND	28,5	None		1	MSS	BAP1, BRCA1, FANCA, KRAS, MAP3K1	None	None
9	F	63	Epithelioid	No	10	1	UND	UND	UND	62,6	BAP1 loss PRKC1 amplification ^#^ TERC amplification ^#^		1	MSS	IDH1, SDHA, ZNF703	None	EZH2 inhibitors
10	M	41	Epithelioid	No	60	UND	No	positive	positive	13,8	TP53 R248W	2,9	UND	UND	JAK2, KMT2A (MLL), MAP2K2 (MEK2), SETD2, TET2	None	None
11	M	40	Sarcomatoid	Yes	60	UND	UND	UND	UND	76,0	NF2 E463* PTEN loss (exons 4–9) CDKN2A/B loss FAS loss	69,3	UND	MSS	ATM, SETD2, TSC2	mTOR inhibitors	FAK inhibitors mTOR inhibitors CDK4/6 inhibitors Pan-ERBB inhibitors AKT inhibitors
12	M	49	Epithelioid	No	5	2	Yes	UND	UND	46,0	BAP1 splice site 35_37 + 2CAGGT > AGGG TERT promotor -124C > T	69,0 6,3	0	MSS	CIC, KDM5A, MLL2, MYCl1, RICTOR, ZNF703	None	EZH2 inhibitors
13	M	52	Epithelioid	No	7,5	UND	UND	UND	UND	50,2	UND	UND	UND	UND	UND	UND	UND
14	M	51	Biphasic	No	20	UND	UND	UND	UND	10,0	BARD1 L479fs*1 CDK12 duplication exon 1	49,0	UND	UND	ARID1A, FAM123B, HSD3B1, KDM5C, PBRM1, RAD51C, ROS1	None	PARP inhibitors
15	M	58	Epithelioid	No	UND	UND	UND	UND	UND	20,0	NF2 L46fs*77	7,6	UND	UND	BRCA2, FGFR3, INPP4B, MPL, PTCH1, ROS1	mTOR inhibitors	FAK inhibitors mTOR inhibitors CDK4/6 inhibitors Pan-ERBB inhibitors
16	M	64	Epithelioid	No	8	1	UND	UND	UND	20,0	ATM E522fs*43	41,9	UND	UND	BAP1, DNMT3A, ESR1, MYCN, NTRK1, POLE	PARP inhibitors	ATR inhibitors PARP inhibitors
17	M	76	Epithelioid	No	5	UND	UND	loss	positive	11,2	ATM V1729fs*20 BAP1 rearrangement intron 10	10,1	UND	UND	ABL1, MSH2, SMO	PARP inhibitors	ATR inhibitors PARP inhibitors EZH2 inhibitors
18	M	71	Epithelioid	No	7,5	UND	UND	UND	UND	26,2	SETD2 R2510fs*2 ^¶^	18,8	0	MSS	ATM, DDR1, ERBB3, LTK, MUTYH, ZNF703	None	None
19	M	53	Epithelioid	Yes	30	UND	UND	loss	positive	35,2	CDKN2A loss WHSC1 E1099K ^¶^	17,5	1	MSS	CTNNB1, MLL2, PARP3, PIM1	None	None
20	M	63	Biphasic	No	5	UND	UND	loss	positive	10,0	SF3B1 K700E	2,1	UND	UND	ALOX12B, APC, CSF1R, mTOR, PDGFRA, SGK1, TEK	None	None

*CPI* checkpoint inhibitor, *IHC* immunohistochemistry, *F* female, *M* male, *MSS* microsatellite stable, *UND* undetermined, *VAF* variant allele frequency,

^a^VAF is calculated as the number of variant reads divided by the number of reads covering the same location and the percentage is estimated based on tumor purity

^b^Approved therapies in the European Union for other tumor types than mesothelioma

^c^Clinical trials that are investigating therapies that targeted genes that were found aberrant in the patient and in which patients with PeM could potentially participate

^d^Additional IHC for MMR proteins showed MLH-1, MSH-2, and PMS-2 proficiency and loss of MSH6

^#^Equivocal copy number alteration, i.e., sequencing data provide some, but not unambiguous, signal that the copy number exceeds the threshold for copy number events assigned to the relevant gene

^♱^Subclonal copy number alteration, i.e., presence of the alteration in < 10% of the assayed tumor DNA

^¶^Sensitivity for the detection of copy number alterations was reduced due to low sample quality

### Genomic signature

**Fig. 1:**
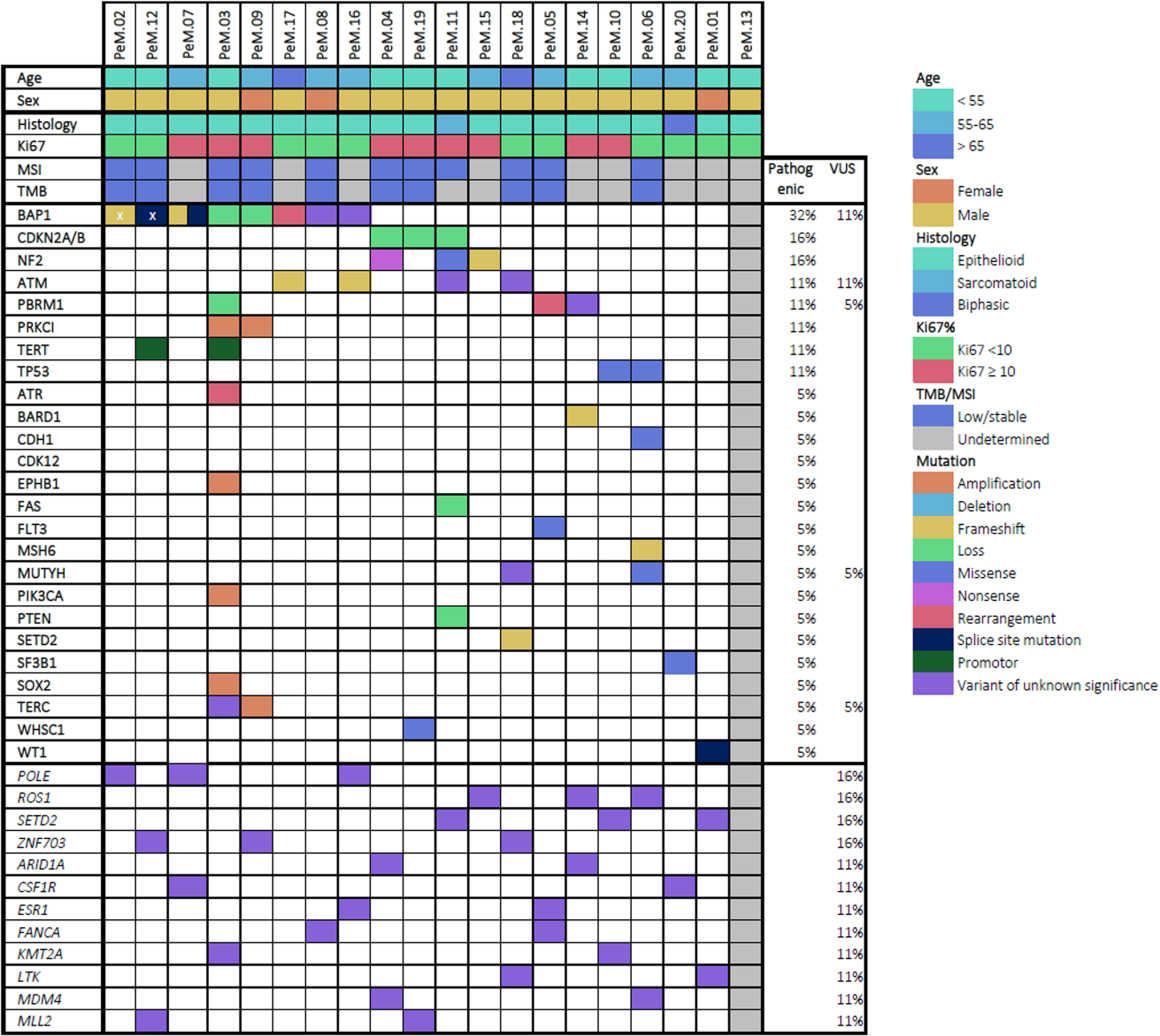
**Mutational landscape of 20 peritoneal mesothelioma (PeM) cases.** x = *BAP1* germline mutation

NGS data were available for 19 samples, as there was one sample failure (Table 1). The TMB could not be determined in nine (47%) cases due to low tumor purity. In all of the remaining cases (*n* = 10), TMB was low (defined as < 10 mutations/Mb). Similar outcomes were observed for MSI, which could not be determined in eight (42%) cases, and the remaining 11 tumors were microsatellite stable (MSS). In one patient, with a MSS tumor according to NGS, a frameshift mutation was detected in mutS homolog 6 (MSH6), encoding for the mismatch repair protein MSH6. Additional IHC for MMR proteins was performed on this sample, showing MLH-1, MSH-2, and PMS-2 proficiency and loss of MSH6 (supplementary Fig. 1). No germline analysis was performed for this patient. The most commonly affected gene in this cohort was *BAP1*, with oncogenic mutations found in six out of 19 patients (32%). In two samples, a variant of unknown significance (VUS) was detected in *BAP1*. Both cyclin dependent kinase inhibitor 2A/B (*CDKN2A/B*) and neurofibromin 2 (*NF2*) harbored mutations in three (16%) tumors. Genes harboring oncogenic mutations in this cohort are depicted in Fig. 1**.** Besides BAP1, *CDKN2A/B*, and *NF2*: ataxia-telangiectasia mutated serine/threonine kinase (ATM), polybromo 1 (*PBRM1*), protein kinase C iota (*PRKCI*), telomerase reverse transcriptase (*TERT*), and tumor protein p53 (*TP53*) were aberrant in ≥ 10% of the sequenced tumors. In Table 2, an overview of all affected genes is provided, including both significant mutations and VUS.

**Table 2:** Overview of mutated genes, number and percentage of affected cases, percentage of VUS

Gene	*N*^a^	%	VUS	%	Gene	*N*^a^	%	VUS	%	Gene	*N*^a^	%	VUS	%	Gene	*N*^a^	%	VUS	%	Gene	*N*^a^	%	VUS	%
*BAP1*	6	32	2	11	*SF3B1*	1	5	2	11	*AR*	0	0	1	5	*JAK2*	0	0	1	5	*PIK3C2B*	0	0	1	5
*CDKN2A/B*	3	16	0	0	*SOX2*	1	5	0	0	*BRCA1*	0	0	1	5	*KDM5A*	0	0	1	5	*PIM1*	0	0	1	5
*NF2*	3	16	0	0	*TERC*	1	5	0	0	*BRCA2*	0	0	1	5	*KDM5C*	0	0	1	5	*PPP2R2A*	0	0	1	5
*ATM*	2	11	2	11	*WHSC1*	1	5	1	5	*CIC*	0	0	1	5	*KDR*	0	0	1	5	*PTCH1*	0	0	1	5
*PBRM1*	2	11	1	5	*WT1*	1	5	1	5	*CTNNA1*	0	0	1	5	*KRAS*	0	0	1	5	*RAD51C*	0	0	1	5
*PRKCI*	2	11	1	5	*POLE*	0	0	3	16	*CTNNB1*	0	0	1	5	*MAP2K2 (MEK2)*	0	0	1	5	*RAR*	0	0	1	5
*TERT* ^b^	2	11	2	11	*ROS1*	0	0	3	16	*CXCR4*	0	0	1	5	*MAP3K1*	0	0	1	5	*RICTOR*	0	0	1	5
*TP53*	2	11	0	0	*ZNF703*	0	0	3	16	*DDR1*	0	0	1	5	*MAP3K13*	0	0	1	5	*SDHa*	0	0	1	5
*ATR*	1	5	0	0	*ARID1A*	0	0	2	11	*DNMT3A*	0	0	1	5	*MEN1*	0	0	1	5	*SGK1*	0	0	1	5
*BARD1*	1	5	0	0	*CSF1R*	0	0	2	11	*EP300*	0	0	1	5	*MPL*	0	0	1	5	*SMO*	0	0	1	5
*CDH1*	1	5	0	0	*ESR1*	0	0	2	11	*ERBB3*	0	0	1	5	*MSH2*	0	0	1	5	*TBX3*	0	0	1	5
*CDK12*	1	5	0	0	*FANCA*	0	0	2	11	*ERRFI1*	0	0	1	5	*MSH3*	0	0	1	5	*TEK*	0	0	1	5
*EPHB1*	1	5	0	0	*KMT2A*	0	0	2	11	*FAM123B*	0	0	1	5	*mTOR*	0	0	1	5	*TERC*	0	0	1	5
*FAS*	1	5	0	0	*LTK*	0	0	2	11	*FGFR3*	0	0	1	5	*MYCI1*	0	0	1	5	*TET2*	0	0	1	5
*FLT3*	1	5	0	0	*MDM4*	0	0	2	11	*GRM3*	0	0	1	5	*MYCN*	0	0	1	5	*TIPARP*	0	0	1	5
*MSH6*	1	5	0	0	*MLL2*	0	0	2	11	*HGF*	0	0	1	5	*NF1*	0	0	1	5	*TSC2*	1	5	2	11
*MUTYH*	1	5	1	5	*ABL1*	0	0	1	5	*HSD3B1*	0	0	1	5	*NOTCH3*	0	0	1	5					
*PIK3CA*	1	5	0	0	*ALK*	0	0	1	5	*IDH1*	0	0	1	5	*NTRK1*	0	0	1	5					
*PTEN*	1	5	0	0	*ALOX12B*	0	0	1	5	*INPP4B*	0	0	1	5	*PARP3*	0	0	1	5					
*SETD2*	1	5	3	16	*APC*	0	0	1	5	*IRF2*	0	0	1	5	*PDGFRA*	0	0	1	5					

^a^Known oncogenic mutations

^b^Promotor mutation

### Variants of unknown significance

Besides known mutations involved in oncogenesis, the F1CDx analysis also provides a report of all VUSes. Variants in polymerase epsilon catalytic subunit (*POLE*), ROS proto-oncogene 1 receptor tyrosine kinase (*ROS1*), and zinc finger protein 703 (*ZNF703*) were determined to be a VUS in 15% of cases each. VUSes that were prevalent in ≥ 10% of cases were also included in Fig. 1. In two samples, a VUS in *BAP1* was detected, resulting in loss of BAP1 expression at IHC.

### Therapy recommendations

The analyses resulted in possible therapy recommendations for five patients (26%). All these recommendations were based on targeted therapies that were approved in the European Union for the treatment of other tumor types. None of these therapies is currently registered as a treatment for mesothelioma. For three (16%) patients with mutations in *NF2*, protein kinase inhibitor (PKI) therapy with either everolimus or temsirolimus could be of interest. For two (11%) other patients, therapy with polyADP-ribose polymerase (*PARP*)-inhibitors might be effective, based on mutations of the *ATM* gene.

### Clinical trials

For patients with mutations in genes for which currently no targeted therapy is available, participation in clinical trials might be beneficial. Based on the NGS data, ten (53%) cases were possibly eligible to participate in clinical trials, based on thirteen observed mutations. Tumors with inactivating mutations, or loss of *BAP1*, are possibly susceptible to treatment with enhancer of zeste homolog 2 (*EZH2*) inhibitors. This resulted in a clinical trial recommendation for six (30%) cases with such a mutation. Two (11%) patients with mutations in *ATM* were possibly eligible to participate in various phase 1 and 2 clinical trials investigating ATR serine/threonine kinase (*ATR*) inhibitors, *PARP* inhibitors and/or DNA-dependent protein kinase catalytic subunit (*DNA-PKcs*) inhibitors. Another two (11%) patients were possibly eligible for participation in various clinical trials targeting focal adhesion kinase (*FAK*), programmed cell death 1 (*PD1*) and mammalian target of rapamycin complex 1/2 (*mTORC1/C2*) based on mutations in *NF2*. Mutations in phosphatase and tensin homolog (*PTEN)* and BRCA1 associated ring domain 1 (*BARD1)* resulted in similar recommendations, involving among others PARP and immune checkpoint inhibition. It should be noted that none of the patients in the current cohort participated in any of these trials, as these trials were not conducted in The Netherlands.

## Discussion

The lack of effective treatments for peritoneal mesothelioma (PeM) makes it interesting to explore the use of targeted therapies that might benefit these patients. Although also rare, pleural mesothelioma is relatively more common and treatment strategies for PeM are commonly derived from the pleural variant. Recently, large cohorts of both pleural and PeM have provided more insights in their mutational profiles and provided possible targets or therapies [7–11, 18]. The mutational profile of the current study cohort is comparable to the TCGA pleural mesothelioma cohort, which is in line with the large cohorts of Hiltbrunner et al. and Dagogo-Jack et al [10, 11, 19].

To evaluate the value of broad NGS in patients with PeM in current practice, we performed broad targeted NGS on tumor biopsies from 20 individual PeM patients. Based on the molecular signature of these tumors, for about one in four patients, potentially effective targeted therapies are available. It should be noted that these targeted treatments have so far not been proven effective against mesothelioma (*pleural or peritoneal*). Therefore, the value of NGS in the current practice for these patients seems limited.

We did identify some clinical trials in which patients with PeM could potentially participate. There are also numerous ongoing trials in other tumor types that are investigating targeted therapies that might be beneficial for patients in our cohort based on the detected aberrations. As new targeted treatments, as well as combination therapies, are being continuously investigated, molecular characterization of individual patient tumors will be increasingly relevant in the future. Below, we reviewed biomarkers generated by NGS that could predict response to certain treatments and the most frequently mutated genes (i.e., oncogenic mutations in ≥ 10% of cases) in the current cohort, for which targeted therapies are currently available.

### TMB and MSI status

TMB was low, and tumors were MSS in all cases for which this could be determined. For one patient in our cohort a MSH6 deficiency was reported. As MSI is a result of a deficient DNA MMR system, MSH6 deficient tumors are per definition MSI. Nonetheless, this tumor was reported as MSS by molecular MSI analysis. Several studies have indicated that molecular MSI analysis has lower sensitivity for MMR deficiency (dMMR) detection compared to IHC, which might be dependent on the origin of the primary tumor; hence, the value of molecular MSI analysis to detect dMMR tumors remains a subject of debate [20, 21]. Likewise, molecular MSI, but also TMB analysis, requires samples with sufficient tumor purity. Low tumor purity is an important challenge to these analyses in daily practice. Panel-based TMB estimation by targeted NGS has been proposed to result in a better estimate of the TMB, compared to the general method of measuring the TMB with the whole exome [22]. Moreover, increasing tumor purity by microdissection is valuable, but unfortunately not possible for send-out FMI tests.

Though MSI and TMB status could not be determined for eight and nine cases, respectively, it is likely that TMB and MSI are mostly low or absent in PeM. Arulananda and colleagues could not identify a single patient with MSI in a cohort of 335 patients with pleural mesothelioma, performed by IHC [23]. There are some studies that reported MSI in patients with mesothelioma, but these cases are rare [10, 24]. With regard to TMB, several studies reported low TMB in the majority of mesothelioma cases (both *pleural and peritoneal)* [10, 11, 25]. As both MSI and high-TMB tumors are associated with a good response to immune checkpoint inhibition (CPI) therapy, one might expect that these therapies are ineffective against mesothelioma [26]. Indeed, the recent checkmate 743 study by Baas et. al showed only modest responses to combination CPI therapy with nivolumab (anti-PD1) and ipilimumab (anti-CTLA4) as a first line treatment for pleural mesothelioma, although long term responders were established [27]. Hence, it is questionable whether MSI and TMB are optimal biomarkers to predict response to CPI.

### Frequently aberrant genes

#### *BAP1*

*BAP1* is the most frequently mutated gene found in mesothelioma (*pleural and peritoneal*), with about 30–50% of cases harboring somatic mutations. (AACR GENIE and COSMIC, February 2022) [28, 29]. Also, a significant proportion of PeM patients might be affected by the so-called ‘*BAP1* tumor predisposition syndrome’ (BAP1-TPDS), as they are carriers of a germline *BAP1* mutation [30]. Besides a predisposition for mesothelioma, these patients are also commonly affected by *BAP1*-inactivated melanocytic tumors, uveal melanoma, cutaneous melanoma and renal cell carcinoma [31]. In line with other studies, we found oncogenic *BAP1* mutations in 32% of tumors in the current cohort, of which two patients were known carriers of a *BAP1* germline mutation [7, 10, 11]. *BAP1* encodes for the tumor suppressor protein ‘ubiquitin carboxyl-terminal hydrolase,’ which plays a role in several cellular processes involved in oncogenesis [32]. Though there are currently no treatments directly targeting *BAP1*, there are therapies targeting molecular pathways in which *BAP1* is involved. *BAP1* is associated with *BRCA1* activation, thereby playing a key role in homologous recombination repair (HRR) [32–34]. Similar to *ATM* deficient tumors, *BAP1* and *BRCA1* deficient tumors might be susceptible to *PARP* inhibition and promising results have been reported in a phase 2 clinical trial [35]. However, in vitro results of sensitivity to *PARP* inhibition and its relationship to *BAP1* status are inconsistent [36–38]. Another potential target is EZH2, which is upregulated in *BAP1* deficient tumors. A preclinical showed increased sensitivity to EZH2 inhibition in *BAP1* deficient mice [39]. A phase 2 trial including 74 patients with BAP1 deficient mesothelioma treated patients with PeM with the EZH2 inhibitor tazemetostat as a monotherapy [40]. A disease control rate of 51% at twelve weeks and 25% at 24 weeks was reported, but no complete and only two partial responses were observed. These modest responses do not seem to be related to BAP1 deficiencies and biomarkers to predict the response to tazemetostat have not yet been identified. Due to its involvement in HRR, BAP1 has also been studied as a biomarker for response to chemotherapy. Wildtype BAP1 has been associated with sensitivity to gemcitabine treatment in mesothelioma cell lines, but this has not been validated in patients with PeM [41, 42].

#### *NF2*

Based on several mutations in *NF2*, protein kinase inhibitors everolimus and temsirolimus could be a potential treatment option for 16% of patients in our cohort. *NF2* is a tumor suppressor gene that plays an important role in cell proliferation and survival [43, 44]. *NF2* is involved in the mammalian target of rapamycin (*mTOR*) signaling pathway. Inactivating mutations of *NF2* lead to cell cycle progression and cell proliferation [45, 46]. *NF2* mutations are reported by previous studies in around 25% of cases of PeM [10, 11]. Some clinical studies and some preclinical evidence suggest that *NF2* inactivation might be associated with response to *mTOR* inhibitors.[47, 48] Everolimus and temsirolimus are both *mTOR* inhibitors and have been approved by the FDA for the treatment of neuroendocrine tumors of the gastro-intestinal tract or lung, HER2/neu-negative breast cancer and renal cell carcinoma, among others. A phase 2 study in *pleural* mesothelioma only showed a 2% response rate to everolimus [49]. This study, however, did not stratify patients based on mutational status. Considering that only about 15% of mesothelioma cases show mutations in *NF2*, the response rate might be higher when only these patients are included. However, some studies suggest that combination treatment might be indicated [50, 51].

#### *ATM*

Mutations in *ATM* were present in two patients in our cohort (11%), but were reported in only 2% of the patients in the large cohort of Hiltbrunner et al. [11]. Although rare, patients with PeM and mutations in *ATM* could benefit from treatment with *PARP* inhibitors. *ATM* is located on chromosome 11 and codes for the *ATM* serine/threonine kinase protein. This protein plays a role in the HRR pathway, among others by p53 activation, which has an important role in cell cycle arrest and apoptosis [52]. Mateo et al. found that deleterious *ATM* mutations in metastatic prostate cancer were associated with good response to olaparib, a *PARP* inhibitor that is approved for the treatment of several solid tumors in the European Union [35, 53]. However, the same group found no survival benefit for castration resistant prostate cancer patients, but these findings were the result of an underpowered interim analysis [54]. For other malignancies, such as gastric-cancer and renal cell carcinoma, similar relations between *ATM* mutations and response to *PARP* inhibition have been reported [55, 56]. Fennell et al. performed a phase 2 trial, treating 26 mesothelioma patients (*25 pleural, 1 peritoneal*) with the *PARP* inhibitor rucaparib after at least one cycle of systemic chemotherapy. They found a disease control rate of 58% at twelve weeks and 23% at 24 weeks, while toxicity was limited [57]. They selected patients with *BAP1* and/or *BRCA1* deficient tumors, other key proteins in HRR. HRR deficient tumors, such as *ATM* inactivated tumors, might have similar responses to PARP inhibition.

### Strengths and limitations

The main strength of this study is the in-depth analysis of PeM molecular characteristics and the evaluation of its value in current daily practice. The current study provides more comprehensive data compared with recently published studies reporting on larger cohorts, which can be valuable for the guidance of future treatment strategies.[10, 11] Though our cohort only included 20 patients, with successful NGS in 19, PeM is such a rare tumor that data of its molecular characteristics remains valuable.

There are some limitations to the current study. As NGS was available for only 20 samples, we selected those patients that were most likely to harbor relevant mutations, resulting in selection bias. In addition, NGS requires sufficient amount of high-quality DNA. For NGS, FMI does not perform any tumor purification, requiring high-quality samples and resulting in a lower sensitivity for the detection of mutations. Selection of high-quality samples might also have resulted in selection bias. Despite this selection, there was one sample failure and TMB/MSI could not be determined in approximately half of the patients due to low tumor purity. This underlines the challenges of NGS in current daily practice, as the success of NGS highly depends on the sample quality and quantity. Despite low tumor purity, we were able to detect relevant mutations in the majority of patients. As the value of TMB/MSI in the treatment of patients with PeM seems limited, low tumor purity might not pose a serious problem in this patient population. Though not a limitation of the current study, another important factor to take into consideration with the interpretation of NGS data is tumor heterogeneity. Tumor heterogeneity results in the possibility of an unrepresentative tumor biopsy, which can be especially relevant in guiding possible treatment choices. Likewise, NGS often identifies variants of unknown significance (VUS), which have no clear clinical implications (yet). For example, one patient in our cohort [8] had a VUS in BAP1, but also showed loss of BAP1 on IHC, making it likely that this is actually a pathogenic mutation. Ongoing research will probably identify the nature of these mutations in the future.

## Conclusion

The value of genomic characterization of PeM tumor samples in daily practice in the Netherlands is currently limited. NGS poses several practical challenges, and effective targeted therapies are limited. For about one in four patients in our cohort, NGS resulted in the identification of potentially effective targeted therapies that are currently available for other tumor types than PeM. Ongoing developments in targeted therapies will result in new treatment options, making genomic characterization increasingly relevant in the future.

## Supplementary Information

Below is the link to the electronic supplementary material.

Supplementary file 1 (DOCX 392 kb)

Supplementary file 2 (TIF 399 kb)

## Data Availability

The data that support the findings of this study are included in the manuscript and any additional data are available from the corresponding author upon reasonable request.
