# Supplementary material for: Genomic characterization and detection of potential therapeutic targets for peritoneal mesothelioma in current practice
Source: Clin Exp Med. 2024 Apr 20;24(1):80. doi: 10.1007/s10238-024-01342-y (PMC11032274; doi:10.1007/s10238-024-01342-y)

**Supplementary data**

**Supplementary table 1. Genes included in FoundationOne CDx panel.**

| ABL1 | BRAF | CDKN1A | EPHA3 | FGFR4 | IKZF1 | MCL1 | NKX2-1 | PMS2 | RNF43 | TET2 |
| --- | --- | --- | --- | --- | --- | --- | --- | --- | --- | --- |
| ACVR1B | BRCA1 | CDKN1B | EPHB1 | FH | INPP4B | MDM2 | NOTCH1 | POLD1 | ROS1 | TGFBR2 |
| AKT1 | BRCA2 | CDKN2A | EPHB4 | FLCN | IRF2 | MDM4 | NOTCH2 | POLE | RPTOR | TIPARP |
| AKT2 | BRD4 | CDKN2B | ERBB2 | FLT1 | IRF4 | MED12 | NOTCH3 | PPARG | SDHA | TNFAIP3 |
| AKT3 | BRIP1 | CDKN2C | ERBB3 | FLT3 | IRS2 | MEF2B | NPM1 | PPP2R1A | SDHB | TNFRSF14 |
| ALK | BTG1 | CEBPA | ERBB4 | FOXL2 | JAK1 | MEN1 | NRAS | PPP2R2A | SDHC | TP53 |
| ALOX12B | BTG2 | CHEK1 | ERCC4 | FUBP1 | JAK2 | MERTK | NT5C2 | PRDM1 | SDHD | TSC1 |
| AMER1 | BTK | CHEK2 | ERG | GABRA6 | JAK3 | MET | NTRK1 | PRKAR1A | SETD2 | TSC2 |
| APC | C11orf30 | CIC | ERRFI1 | GATA3 | JUN | MITF | NTRK2 | PRKCI | SF3B1 | TYRO3 |
| AR | CALR | CREBBP | ESR1 | GATA4 | KDM5A | MKNK1 | NTRK3 | PTCH1 | SGK1 | U2AF1 |
| ARAF | CARD11 | CRKL | EZH2 | GATA6 | KDM5C | MLH1 | P2RY8 | PTEN | SMAD2 | VEGFA |
| ARFRP1 | CASP8 | CSF1R | FAM46C | GID4 (C17orf39) | KDM6A | MPL | PALB2 | PTPN11 | SMAD4 | VHL |
| ARID1A | CBFB | CSF3R | FANCA | GNA11 | KDR | MRE11A | PARK2 | PTPRO | SMARCA4 | WHSC1 |
| ASXL1 | CBL | CTCF | FANCC | GNA13 | KEAP1 | MSH2 | PARP1 | QKI | SMARCB1 | WHSC1L1 |
| ATM | CCND1 | CTNNA1 | FANCG | GNAQ | KEL | MSH3 | PARP2 | RAC1 | SMO | WT1 |
| ATR | CCND2 | CTNNB1 | FANCL | GNAS | KIT | MSH6 | PARP3 | RAD21 | SNCAIP | XPO1 |
| ATRX | CCND3 | CUL3 | FAS | GRM3 | KLHL6 | MST1R | PAX5 | RAD51 | SOCS1 | XRCC2 |
| AURKA | CCNE1 | CUL4A | FBXW7 | GSK3B | KMT2A (MLL) | MTAP | PBRM1 | RAD51B | SOX2 | ZNF217 |
| AURKB | CD22 | CXCR4 | FGF10 | H3F3A | KMT2D (MLL2) | MTOR | PDCD1 | RAD51C | SOX9 | ZNF703 |
| AXIN1 | CD274 | CYP17A1 | FGF12 | HDAC1 | KRAS | MUTYH | PDCD1LG2 | RAD51D | SPEN |  |
| AXL | CD70 | DAXX | FGF14 | HGF | LTK | MYC | PDGFRA | RAD52 | SPOP |  |
| BAP1 | CD79A | DDR1 | FGF19 | HNF1A | LYN | MYCL | PDGFRB | RAD54L | SRC |  |
| BARD1 | CD79B | DDR2 | FGF23 | HRAS | MAF | MYCN | PDK1 | RAF1 | STAG2 |  |
| BCL2 | CDC73 | DIS3 | FGF3 | HSD3B1 | MAP2K1 | MYD88 | PIK3C2B | RARA | STAT3 |  |
| BCL2L1 | CDH1 | DNMT3A | FGF4 | ID3 | MAP2K2 | NBN | PIK3C2G | RB1 | STK11 |  |
| BCL2L2 | CDK12 | DOT1L | FGF6 | IDH1 | MAP2K4 | NF1 | PIK3CA | RBM10 | SUFU |  |
| BCL6 | CDK4 | EED | FGFR1 | IDH2 | MAP3K1 | NF2 | PIK3CB | REL | SYK |  |
| BCOR | CDK6 | EGFR | FGFR2 | IGF1R | MAP3K13 | NFE2L2 | PIK3R1 | RET | TBX3 |  |
| BCORL1 | CDK8 | EP300 | FGFR3 | IKBKE | MAPK1 | NFKBIA | PIM1 | RICTOR | TEK |  |

**Supplementary figure 1: Immunohistochemical staining for MSH-2, MSH-6, MLH-1, and PMS-2.**


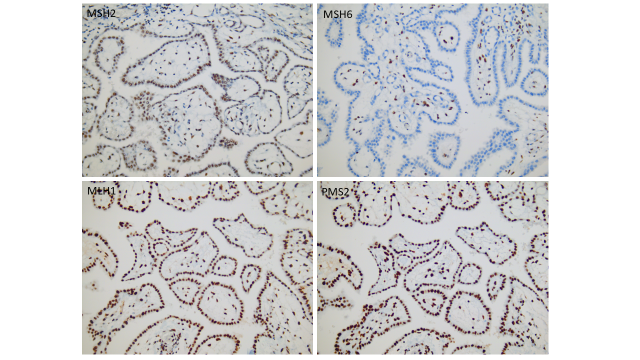

Supplement: Supplementary file 1 — (DOCX 392 kb) [file 10238_2024_1342_MOESM1_ESM.docx]
