# Supplementary figures and images for: Genomic characterization and detection of potential therapeutic targets for peritoneal mesothelioma in current practice
Source: Clin Exp Med. 2024 Apr 20;24(1):80. doi: 10.1007/s10238-024-01342-y (PMC11032274; doi:10.1007/s10238-024-01342-y)

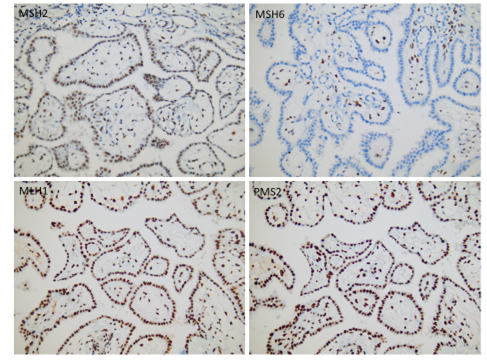

Supplement: Supplementary file 2 — (TIF 399 kb) [file 10238_2024_1342_MOESM2_ESM.tif]
